# The oldest known bat skeletons and their implications for Eocene chiropteran diversification

**DOI:** 10.1371/journal.pone.0283505

**Published:** 2023-04-12

**Authors:** Tim B. Rietbergen, Lars W. van den Hoek Ostende, Arvid Aase, Matthew F. Jones, Edward D. Medeiros, Nancy B. Simmons

**Affiliations:** 1 Naturalis Biodiversity Center, Leiden, The Netherlands; 2 Fossil Butte National Monument, Kemmerer, Wyoming, United States of America; 3 Division of Vertebrate Paleontology, Department of Ecology and Evolutionary Biology, Natural History Museum and Biodiversity Institute, The University of Kansas Lawrence, Lawrence, Kansas, United States of America; 4 Boreal Ecosystems and Agricultural Sciences, Grenfell Campus, Memorial University of Newfoundland, Corner Brook, Newfoundland, Canada; 5 School of Biology and Ecology, University of Maine, Orono, Maine, United States of America; 6 Division of Vertebrate Zoology, Department of Mammalogy, American Museum of Natural History, New York, New York, United States of America; Ecole Normale Supérieure de Lyon, FRANCE

## Abstract

The Fossil Lake deposits of the Green River Formation of Wyoming, a remarkable early Eocene Lagerstätte (51.98 ±0.35 Ma), have produced nearly 30 bat fossils over the last 50 years. However, diversity has thus far been limited to only two bat species. Here, we describe a new species of *Icaronycteris* based on two articulated skeletons discovered in the American Fossil Quarry northwest of Kemmerer, Wyoming. The relative stratigraphic position of these fossils indicates that they are the oldest bat skeletons recovered to date anywhere in the world. Phylogenetic analysis of Eocene fossil bats and living taxa places the new species within the family Icaronycteridae as sister to *Icaronycteris index*, and additionally indicates that the two Green River archaic bat families (Icaronycteridae and Onychonycteridae) form a clade distinct from known Old World lineages of archaic bats. Our analyses found no evidence that *Icaronycteris*? *menui* (France) nor *I*. *sigei* (India) belong to this clade; accordingly, we therefore remove them from Icaronycteridae. Taken in sum, our results indicate that Green River bats represent a separate chiropteran radiation of basal bats, and provide additional support for the hypothesis of a rapid radiation of bats on multiple continents during the early Eocene.

## Introduction

With over 1,400 living species, bats are the second most speciose group of mammals [1, 2]. They are found all over the world with the exception of polar regions and a few remote islands, and are ecologically highly diverse, occupying a wide variety of habitats and ecological niches [1, 3]. Bats play a vital role in healthy natural ecosystems and additionally provide many ecosystem services important for human economies (e.g. pest control, pollination, seed dispersal) [4–11]. Bats are also the only mammals capable of true powered flight, an adaptation that evolved early in the history of the chiropteran lineage [12].

The earliest confirmed records of bats are from early Eocene (Ypresian; 56–47.6 Ma) deposits [13]. During the Ypresian, bats reached a nearly global distribution, being found in deposits from North America [12, 14, 15], Europe [16–21], Africa [22–25], Asia [26–28], and Australasia [29–31]. The early Eocene is known for an extreme rise in temperature known as the Palaeocene-Eocene Thermal Maximum (PETM) and the early Eocene Climate Optimum (EECO) [32, 33], and additionally for an explosive radiation of mammals, insects, and flowering plants [34–36]. Despite having a fossil record comprising more than 70 different taxa from the Eocene, early bat evolution remains relatively poorly understood [13, 37]. Complete fossil bat skeletons are rare, and the majority of the bat fossil record consists of isolated teeth [16, 18, 38, 39]. However, more complete remains of bats occur at several Eocene localities in North America, Europe, and Africa. The oldest known articulated bat skeletons come from mid-shore deposits of the Green River Formation in southwestern Wyoming (± 52.5 mya). Two fossil bat species have been described previously from the Green River Formation: *Icaronycteris index* Jepsen, 1966 and *Onychonycteris finneyi* Simmons et al., 2008. Both of these taxa are known from nearly complete skeletons. These fossil bats were all discovered in the Fossil Lake Sediments, deposited by the smallest lake of the Green River lake system [40]. So far, no Green River bats have been described from the other lakes of the Green River Formation (Lake Gosiute and Lake Uinta). The lowlands of the Fossil Lake basin were warm and humid, similar to a subtropical environment. This basin was surrounded by highlands and mountains, where a more temperate highland flora could be found [41].

Given the very high taxonomic and functional diversity of living bat faunas today [2, 42–45], it would not be surprising if the area and habitats reflected in the Green River Formation hosted more bat species than previously discovered. Here, we describe a new species of bat from the Green River Formation, representing an increase of 50% in the known bat diversity.

## Material and methods

No permits were required for the described study, which complied with all relevant regulations. We examined multiple specimens of Eocene fossil bats (S1 Appendix), as well as literature descriptions of all Eocene bats known from skeletons, and evaluated osteological characters including, but not restricted to, those discussed by Jepsen [14], Simmons and Geisler [37], Simmons et al. [12] and Smith et al. (2012). Dental homology nomenclature for premolars follows that of Simmons and Geisler [37], considering the missing elements in the premolar row to represent the P2 and p2 in the upper and lower dentition, respectively.

CT scans of the holotype of the new species were made using a high-resolution Phoenix v|tome|x micro-CT scanner in the Microscopy and Imaging Facility at the American Museum of Natural History. Visualization (S1 Fig) and analyses were performed in VGStudio MAX 3.0.2 and Avizo 9.5.0. Measurements of the new fossil bat were obtained from micro-CT images. Each measurement was taken three times and an average was taken to minimize measuring error. All measurements reported herein are from adult individuals with closed epiphyses. Measurements (in mm) are in S1 Table.

To estimate body mass, we followed the methods described by Giannini et al. [46]. Body mass M (in grams) was estimated from least mid-shaft diameter data of the humerus (D) for fossil specimens. As recommended by Giannini et al., we transformed the equation: log10 (M) = (log10(D) − log10(b0))/b1 into a general model: log10 D = log10 b0 + b1 * log10 M + error, where b0 is the y-intercept and b1 is the slope parameter of the corresponding equation from extant bats (S1 File). Giannini et al. [46] showed that the least mid-shaft diameter of the humerus best explained the model (r^2^ = 0.991) across a wide range of bat taxa with the corresponding values: b0 = -0.273; b1 = 0.363.

We conducted a phylogenetic analysis in order to determine the relationships of the new species of *Icaronycteris* to other species currently placed in the family Icaronycteridae as well as other well-known Eocene bats. Our morphology-based parsimony analysis included six other Eocene bat species known from complete skeletons (*Icaronycteris index*, *Onychonycteris finneyi*, *Archaeonycteris trigonodon*, *Palaeochiropteryx tupaiodon*, *Hassianycteris messelensis*, and *Tachypteron franzeni*), and the two other named species of *Icaronycteris* known only from the dentition (*I*.? *menui* and *I*. *sigei*). Extant bats representing living superfamilies (*Saccopteryx bilineata*, Emballonuroidea; *Macrotus waterhousii*, Noctilionoidea; *Myotis lucifugus*, Vespertilionoidea; *Rhinolophus ferrumequinum* and *Rhinopoma hardwickii*, Rhinolophoidea) were included in order to determine the relationship of the fossil taxa to crown Chiroptera. *Erinaceus europaeus* and *Sorex bendirii* (Eulipotyphla) were included as outgroups representing non-Chiroptera Laurasiatheria.

A total of 568 discrete characters (248 craniodental characters and 320 postcranial characters) were scored as much as possible for each taxon. Completeness of taxon scoring ranged from a high of 95% (in extant species) to a low of 14% (in the incomplete fossil taxon *Icaronycteris sigei*). Multistate characters were treated as ordered only when clear intermediate states existed (e.g., i1 crown proportions: width greater than length; length and width subequal; length greater than width). A molecular backbone was used to constrain the relationships of living taxa following the relationships recovered by Amador et al. [47]. The dataset was compiled in Morphobank [48] and is available there as Project P4016. When possible, characters were scored from direct observation of specimens (S1 Appendix), otherwise characters were scored from the literature. Phylogenetic analyses were conducted in PAUP* version 4.0a169 [49], using a non-heuristic, exact search (i.e., branch-and-bound) to produce the most parsimonious trees. Due to the unstable position of the most fragmentary fossil taxa (i.e., *Icaronycteris*? *menui* and *I*. *sigei*) in initial analyses, a second analysis was conducted using a backbone tree constraining the relationships of the extant bats and fossil taxa known from complete skeletons.

Institutional abbreviations: American Museum of Natural History, New York [Fossil Mammals] (AMNH:FM); Houston Museum of Natural Sciences (HMNS); Field Museum of Natural History, Chicago (FMNH); Fossil Butte National Monument (FOBU); Royal Ontario Museum, Toronto (ROM); Wyoming Dinosaur Center, Wyoming (WDC); The University of Wyoming Department of Geology and Geophysics Collection of Fossil Vertebrates (UW); Yale Peabody Museum, former Princeton University collections (YPM-PU).

## Systematic paleontology

Order Chiroptera Blumenbach, 1799

Family Icaronycteridae Jespen, 1966

Genus *Icaronycteris* Jepsen, 1966

*Icaronycteris gunnelli* sp. nov.

### Holotype

**Fig 1:**
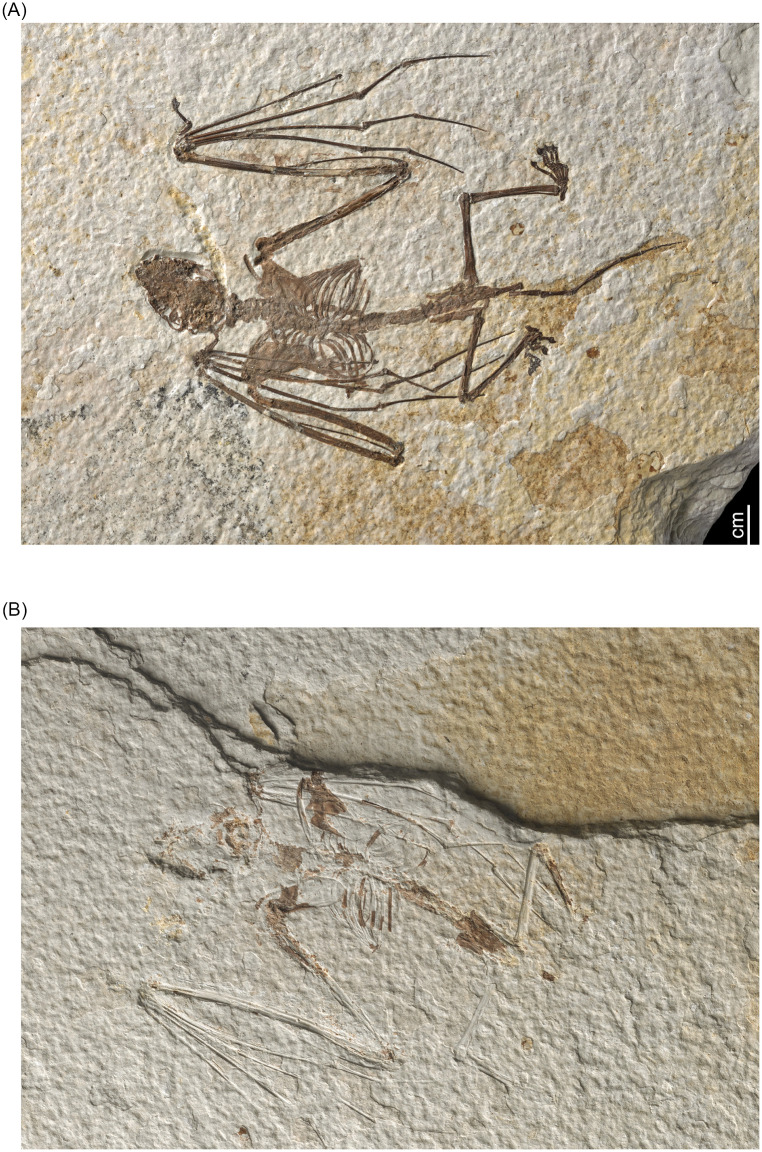
Skeleton of Holotype of *Icaronycteris gunnelli* (FM.145747A) A) Dorsal view; B) Counterpart (FM.145747B).

**Fig 2:**
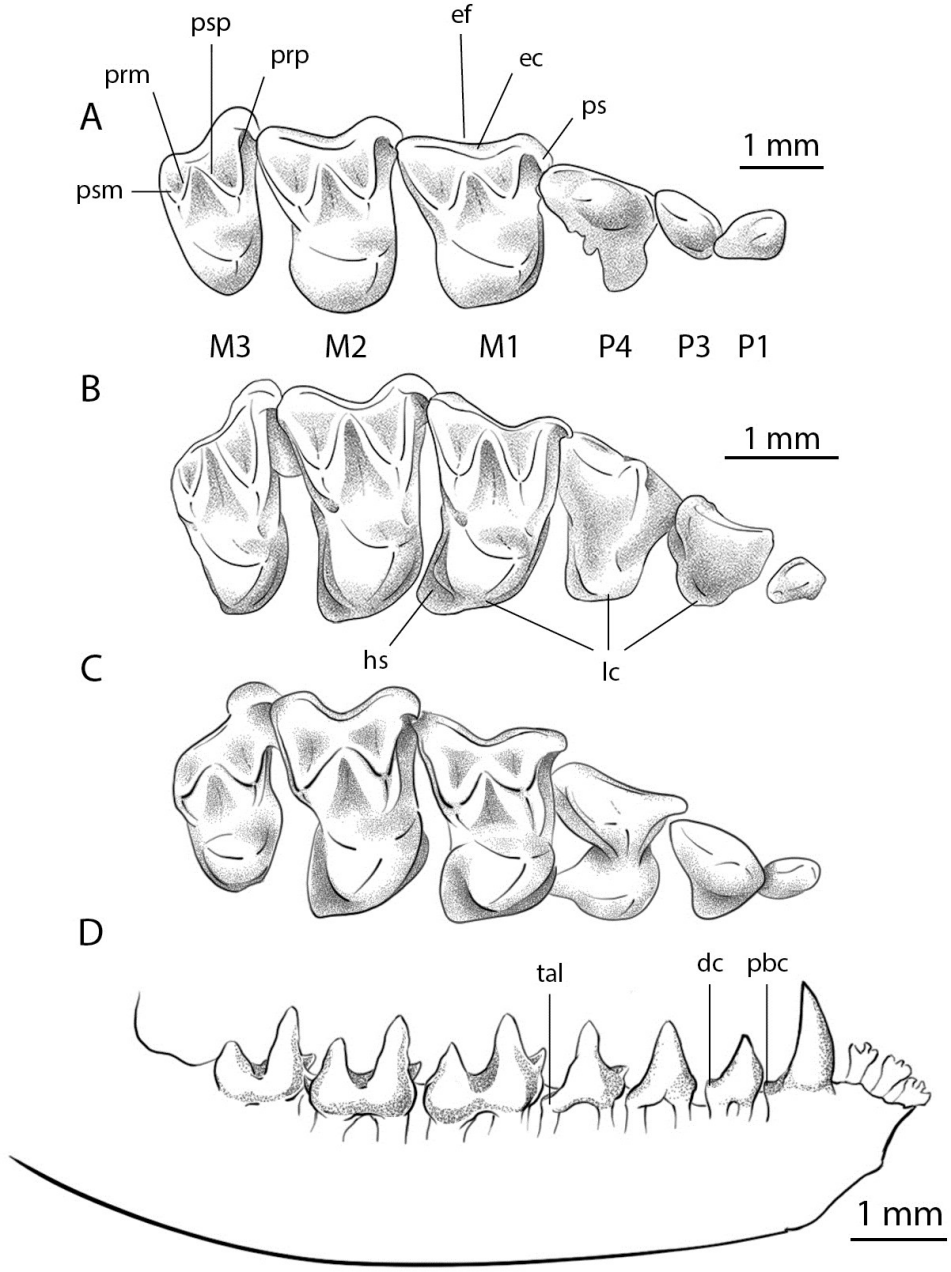
Illustrated dentitions of Green River bats. A) *Onychonycteris finneyi* (ROM 55351A-B) upper P1-M3; B) *Icaronycteris index* (YPM-PU 18150) upper P1-M3; C) Icaronycteris gunnelli (FM.145747A) upper P1-M3; D) *Icaronycteris gunnelli* (FM.145747A) lower c1-m3, with reconstructed i1-i3, in labial view. Abbreviations of features discussed in the text: dc—distal cingulid; ec—ectocingulum; ef—ectoflexus; hs—hypocone shelf; lc—lingual cingulum; pbc—posterobasal cusp; prm—premetacrista; prp—preparacrista; ps—parastyle; psm—postmetacrista; psp—postparacrista; tal—talonid.

AMNH:FM:145747A,B (part and counterpart), an articulated skeleton including skull and mandibles with restoration (Figs 1 and 2).

### Paratype

**Fig 3:**
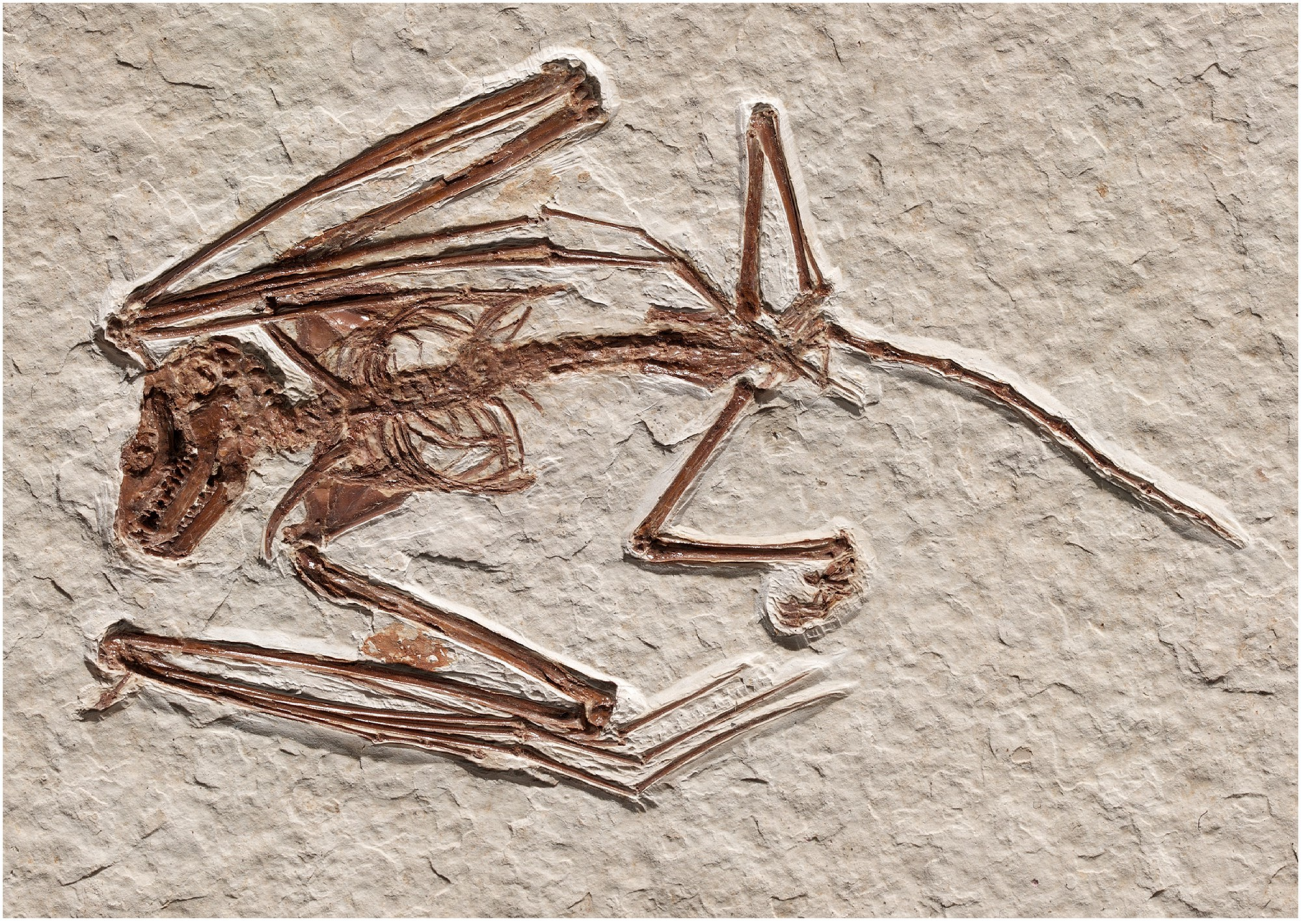
Skeleton of paratype of *Icaronycteris gunnelli* (ROM:Palaeobiology-Vertebrate Fossils:52666).

ROM:Palaeobiology-Vertebrate Fossils:52666, an articulated skeleton including skull and mandibles (Fig 3).

### Locality, horizon, and age

**Fig 4:**
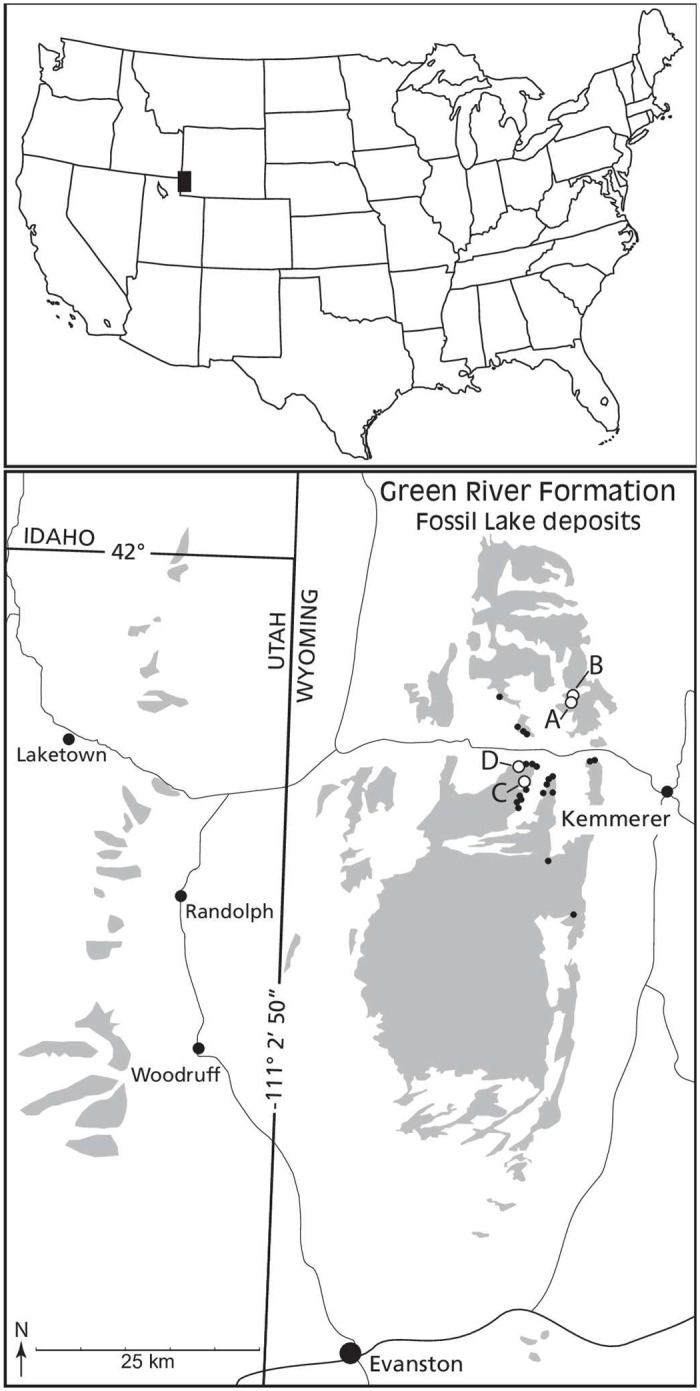
Remaining deposits of Fossil Lake sediments of the Green River Formation are illustrated in gray with highly-fossiliferous, deep-water, laminated limestone deposits of Eocene Fossil Lake in southwest Wyoming and poorly-fossiliferous, shallow-water, non-laminated limestones in northeast Utah and southeast Idaho. Eleven of more than 20 fossil quarries are still active today. Three active quarries (A, B and D) and one inactive quarry (C) have produced fossil bats currently held in public institutions. All other historic and active quarry locations are marked by small black dots. American Fossil Quarry (A) yielded *I*. *gunnelli* (AMNH.FM.145747 and ROM.52666), I. index (AMNH.FM.125000 and AMNH.FM.144215), *I*. cf *index* (WDC-CGR-115) and O. finneyi (AMNH.FM.142467 and ROM.55351). Thompson Ranch north quarry (B) yielded *I*. *index* (FMNH.PM.62096 and HMNS.PV.001468). The Holland brother’s quarry (C) was active for only one season in the mid-1930s and yielded *I*. *index* (YPM-PU.18150). The Smith Hollow quarry (D) yielded a poorly preserved and unidentified specimen (FOBU13777). Most bat specimens were recovered from two quarries operating continuously since the 1980s in nearshore deposits. A combination of proximity to the eastern shore and a large volume of rock excavated is the most probable explanation for the greater number of bat fossils being discovered in those locations.

**Fig 5:**
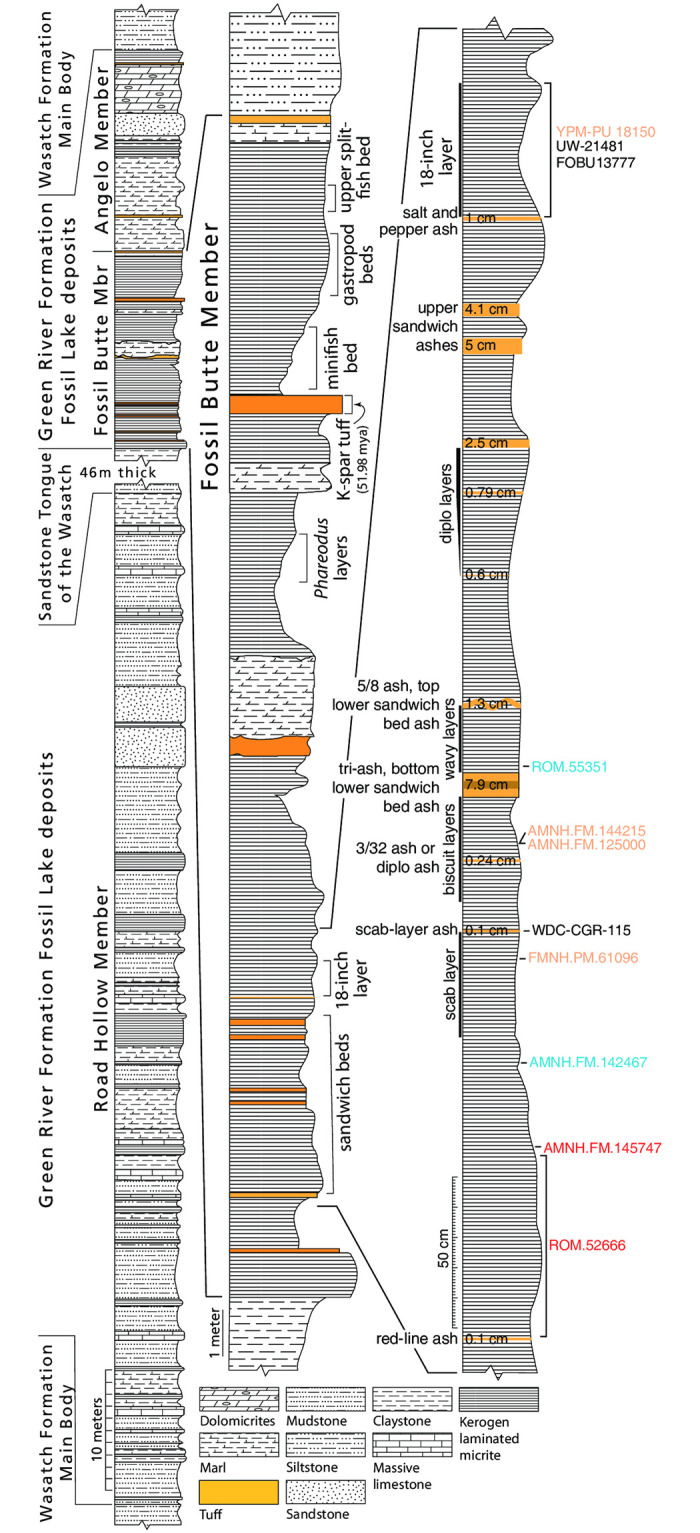
A composite stratigraphic column of the Green River Formation showing the relative position of the Fossil Butte Member and stratigraphic levels where various bat fossils have been found. The Fossil Butte Member is the thinnest of the members in the Green River Formation, but was deposited when the lake was deepest, providing conditions that were optimal for exceptional preservation. Five units in the Fossil Butte Member (Sandwich Beds, 18-inch Layer, Minifish Bed, Gastropod Beds, and Upper Split-fish Bed) are actively quarried on private and state-owned land by companies collecting the abundant fossil fish to sell. Among the fish, one bat is found on average every two years. To date, all bats have come from the 18-inch Layer and Sandwich Beds. Bat specimens are coded by color: *Icaronycteris gunnelli* in red, *I*. *index* in orange, *Onychonycteris finneyi* in blue, and unidentified or indeterminate are black. More than 10 bat specimens are held in private collections (not shown here), but none are confirmed lower in the section than the two specimens of *I*. *gunnelli*. Left two columns modified after Buchheim et al, 2011 [40].

The holotype was found by Terry Rickords on August 16, 2017 at the American Fossil Quarry (previously known as the Thompson Ranch South Quarry or (South) Dempsey Quarry), Lincoln County, Wyoming (N41°51.872’ W 110°40.491’), ± 1,15 meter below the Tri-ash layer in the sandwich beds of the Fossil Butte Member (FBM) (Figs 4 and 5), Green River Formation (GRF), late early Eocene (Lostcabinian, Wasatchian Biochron Wa-7). This locality preserves near-shore lacustrine deposits of Fossil Lake [40, 50]. The volcanic ash layer near the top of the FBM is radiometrically dated at 51.98 ± 0.34 Ma [51, 52]. Deposition rates are unknown in Fossil Lake. Deposition near-shore is more rapid than mid-lake [53], confounding efforts to estimate rates of deposition. Until reliable deposition rates are established there is no reliable means to estimate ages of specimens. The paratype was found in the same quarry as the holotype by Robert Kronner in 1994 in the bottom 60 cm of the sandwich beds of the FBM, Green River Formation.

### Etymology

This species name is in honor of Gregg Gunnell in recognition of his extensive contributions to the understanding of fossil bats and chiropteran evolution.

### Diagnosis

*Icaronycteris gunnelli* is the smallest known chiropteran from the Green River Formation (S1 Table) and is distinguished from other Eocene bats by the following combination of traits: claw present on wing digit I and II; tiny ossified third phalanx present on wing digit III–V; relatively short forearm and broad wing; relatively short, robust hind limbs with a possibly sutured tibia and fibula; lower canine tall and lanceolate; upper canine with anteromedial groove; diastema present between C and P1; P3 with raised lingual cingulum and a crown length relatively shorter than that of P4; P4 with well-developed lingual cingular cusp and labial cingulum; P4 length and width subequal; upper molars with strongly developed ectocingulum.

### Description and comparisons

The skull of the holotype of *Icaronycteris gunnelli* is dorsoventrally crushed, so many cranial features, including structures of the basicranium, cannot be distinguished. However, the shape of the skull and several important traits of the skull and postcranium can be evaluated. Morphological traits of *I*. *gunnellii* are similar to those seen in *Icaronycteris index* and *Onychonycteris finneyi* except if explicitly stated otherwise below. The proclivous premaxilla in *Icaronycteris gunnelli* extends anteriorly beyond the level of the canine root and the nasal process of the premaxilla is well developed. The zygomatic arch appears complete and is broader than the mastoid region. Parietals appear to have not been inflated based on their rounded shape. The angular process of the dentary in *I*. *gunnelli* projects below the level of the occlusal plane, and the coronoid process is approximately two times the height of the condylar process.

The dental formula of *I*. *gunnelli* is I2/3, C1/1, P3/3, M3/3 = 38. The upper incisors are orthodont and orthoclivous, and in this they resemble those of *Onychonycteris finneyi*. In contrast, the upper incisors in *I*. *index* are proodont and orthoclivous. The crowns of the upper incisors are not clearly differentiated from the root shafts in *I*. *gunnelli*, a condition similar to *I*. *index* but different from that seen in *O*. *finneyi*, in which the crown and root shaft of the upper incisors are clearly differentiated. The occlusal margin of I1 includes a large main cusp that is offset mesially from the axis of the tooth and tapers to a blunt point. A distal accessory cusp is present lateral to the main cusp on I1, with the accessory cusp approximately ¼ the size of the main cusp. The two upper incisors are subequal in height. Crown morphology of I2 is well developed and lingual cingulum is absent. Lack of lingual cingulum of the I2 is a characteristic also seen in *I*. *index*, whereas a lingual cingulum is present on this tooth in *O*. *finneyi*. I1 and I2 are subequal in height in *I*. *gunnelli*, also a condition shared with *I*. *index*. In contrast, in *O*. *finneyi* the height of I1 is less than that of I2. The upper canine (C) is separated from I2 by a diastema in all of these taxa.

The upper canine in *I*. *gunnelli* is elongated anteroposteriorly but does not project anteriorly. This tooth is not labially swollen and it lacks a raised cingulum or accessory cusp. No lingual cingulum is present, which is another trait shared with *I*. *index*. By contrast, *O*. *finneyi* exhibits a faint lingual cingulum on the upper C. The distal cingulum of the upper canine is small in *I*. *gunnelli*. A labial cingulum on the canine was not visible on the X-ray, but we could not determine if this indicated true absence or a lack of resolution in our images. An anteromedial groove is present on the upper canine, a structure that is not present in *I*. *index* or *O*. *finneyi*. A posteromedial ridge is present and the posterolingual and posterolabial surfaces of the canine are flattened. The upper canine has a tiny posterolateral accessory cusp, a structure that is absent in *O*. *finneyi*, and present but slightly smaller in *I*. *index*. The shape of this cusp is conical and is not clearly separated from the main cusp; it originates on the posterior face of the main cusp. A diastema is present between upper C and P1.

The upper premolars lie in line with the molar toothrow and no teeth are offset medially or laterally. The P1 is single rooted. This tooth has a well-developed crown which lacks a labial cingulum, similar to *I*. *index* but differing from *O*. *finneyi*, which has a labial cingulum on P1. The P3 in *I*. *gunnelli* also lacks a labial cingulum, again as in *I*. *index*, with both *Icaronycteris* species differing from *O*. *finneyi*, which exhibits a weakly developed labial cingulum on P3. The lingual cingulum of P3 is weakly developed and lacks a cusp, but the edge of the cingulum is slightly raised. This differs from the condition in *I*. *index* and *O*. *finneyi*, both of which have a lingual cusp on P3. The crown length of P1 is shorter than that of P3. This condition is shared with *I*. *index*, while P1 and P3 are subequal in crown length in *O*. *finneyi*. The postparacrista of P3 extends as a single crest to the distal edge of the tooth and lacks a distal accessory cusp. Both P3 and P4 have three roots, but the crown length of P3 is shorter than that of P4. The latter trait contrasts with the condition in *I*. *index* and *O*. *finneyi*, both of which have a P3 that is longer than P4. The height of P3 is subequal to that of P4 in *I*. *gunnelli*. This differs from both *I*. *index*, which has a P3 that is taller than P4, and from *O*. *finneyi*, in which P3 is shorter in height than the P4. No diastema present between the P3 and P4. The lingual cingulum of the P4 is very large and resembles that of *I*. *index*. In both of these taxa, the lingual cingulum forms a distinct lobe that extends posteriorly as far as the protocone of the first molar. This condition differs from that seen in *O*. *finneyi*, where the lobe of the lingual cingulum does not extend as far as the protocone of the first molar. The labial cingulum of the P4 is well developed in *I*. *gunnelli* and *O*. *finneyi*, but absent in *I*. *index*. The P4 in *I*. *gunnelli* has three roots. The lingual cingulum of the P4 is very large and resembles that of *I*. *index*. In both of these taxa, the lingual cingulum forms a distinct lobe that extends posteriorly as far as the protocone of the first molar. This condition differs from that seen in *O*. *finneyi*, where the lobe of the lingual cingulum does not extend as far as the protocone of the first molar. The labial cingulum of the P4 is well developed in *I*. *gunnelli* and *O*. *finneyi*, but absent in *I*. *index*. A lingual cingular cusp is well developed on P4 in *I*. *gunnelli*. This differs from the condition in *I*. *index*, in which the lingual cingular cusp is weakly developed. The condition of a lingual cingular cusp in *O*. *finneyi* cannot be determined from the specimens available. The P4 postparacrista is a single crest that extends as a continuous crest to the distal aspect of the tooth. The length and width of the P4 are subequal in *I*. *gunnelli*, in contrast to the conditions seen in *I*. *index* (P4 wider than long) and *O*. *finneyi* (P4 longer than wide).

The upper molars of *I*. *gunnelli* are characterized by a strongly developed ectocingulum. This is similar to the condition seen in *O*. *finneyi*, but differs from that in *I*. *index*, in which the ectocingulum is present but weak. A lingual cingulum and stylar shelf are present on both the M1 and M2, and the protocone and paracone are subequal in height. The protocone on both M1 and M2 is sharp and inclined anteriorly. The angle between the postparacrista and premetacrista is acute. The postparacrista contacts the premetacrista on the labial aspect of the tooth and closes the trigon basin labially on both M1 and M2. The trigon basin on both teeth is wide and shallow, and a mesostyle and mesostylar crest are absent. The contact between the postparacrista and premetacrista is more lingually displaced than the condition observed in *I*. *index* and similar to that of *O*. *finneyi*, and both M1 and M2 have a cuspule on the premetacrista as well as a small paraconule. The maximum mesiodistal length of M1 and M2 is less than half the distance between the paracone and metacone of the respective teeth.

The M1 has a long, straight, crestlike, parastyle that is not separated from the preparacrista. A single deep ectoflexus is present labial to the mesostyle. The preparacrista of M1 is subequal in length to the postparacrista, and the postmetacrista is about 1.5 times longer than the premetacrista. The trigon basin does not show any striations or enamel folds, instead being relatively smooth. The postprotocrista is oriented distolabially towards the metacone, and it extends labially to become confluent with the metacingulum and the edge of the hypocone shelf. A metaconule is present on the M1, but an endoloph is absent. The hypocone shelf is small and narrow but clearly distinct from the trigon basin. The M2 parastyle is crestlike and curved. Like M1, the M2 has a single deep exoflexus labial to the mesostyle. The metacone is located directly posterior to the paracone. The M2 postmetacrista, which is oriented distolabially towards the metacone, is 1.75 times longer than the premetacrista. An endoloph is present on M1, but is lacking on M2. The hypocone shelf on the M2 is very small and appears only as a narrow cingulum; no hypocone cusp is present.

The M3 is moderately reduced with a surface area <75% that of M2. The anterior segment of the M3 is nearly as large as that of the M2, and a long, crested curved parastyle and a paraconule are present. The premetacrista and postparacrista of M3 are subequal in length, and the metacone is present as a distinct cusp. A metastylar fovea is lacking on the M3, but the protocone and small hypocone shelf are present.

The three lower incisors in *I*. *gunnelli* are trilobed and somewhat procumbent; they are all similar in size and shape. The alveoli are evenly spaced, and there is no diastema between the last incisor and lower canine. The lower canine is tall, lanceolate, and lacks an anterior cuspule. The posterior cingulid on the canine is broad and a posterobasal cusp is strongly developed. The labial cingulum and cingular structures of the canine are not clearly visible given preservation of the holotype of *I*. *gunnelli* and hence cannot be evaluated.

As in other Eocene bats, *I*. *gunnelli* has three lower premolars. The p1 is large, single-rooted, cuspidate and has a distal cuspule. The crown length of p1 is less than that of p3. This is similar to the condition seen in *I*. *index*, but different from that of *O*. *finneyi*, in which p1 crown length is greater than p3 length. The p3 is premolariform, i.e., the cusps are not arranged in a tribosphenic pattern. Anterolingual and distal cuspules are present, but there is no lingual cuspule. Both p3 and p4 have two roots that are oriented longitudinally with respect to the long axis of the tooth row. The crowns of p3 and p4 are subequal in height and length but differ in morphology; in contrast to p3, the p4 is tribosphenic with a distinct trigonid and talonid. The paraconid of p4 consists of a single large cusp supporting a paracristid; this tooth also has a large metaconid, a long protocristid, and a long talonid. The protoconid is tall, sharp and slender. Labial structures on the premolars are not visible and therefore cannot be described in detail.

The lower molars of *I*. *gunnelli* lack a lingual cingulid and the trigonid fovea is open lingually. The hypoconulid is located on the distolingual border of the talonid, well separated from the entoconid and closer to the midline than to the entoconid. The height of the lingual cusps appears relatively low compared to the labial cusps. The entocristid is disrupted (the entocristid is short so that talonid basin drains lingually) on all three lower molars. The m1 and m2 are subequal in size and have similar crown morphology including a well-developed protoconid, paraconid, and metaconid. *I*. *gunnelli* and *I*. *index* both have a large hypoconulid on the m1 and m2, but in *O*. *finneyi* the hypoconulids on these teeth are small and low. The protoconid on m1 is markedly higher than the hypoconid, but is subequal in height to the protoconids on the m2 and m3. The m3 has two roots and bears a large hypoconulid.

The axial skeleton of *I*. *gunnelli* generally resembles that of other Eocene bats. An accurate vertebral count in the thoracolumbar region could not be determined due to poor preservation. The tail has 11 free caudal vertebrae. The scapula has an acromion process that extends anterolaterally beyond the glenoid fossa, and the lateral and dorsal edges of the acromion are flat. The infraspinous fossa is narrow and triangular shaped, tapering anteriorly towards the glenoid. A thick lip is present along the axillary border of the scapula. The coracoid process is short, stout, and appears to curve ventrolaterally.

Like other known Eocene bats, *I*. *gunnelli* has a well-developed wing skeleton with elongated hand and finger bones. The relative length of the humerus compared to the radius is ~76%, which is comparable to that of *O*. *finneyi* (~76%) while somewhat greater than that of *I*. *index* (~71%). The head of the humerus is spherical, and the distal articular surface of the humerus is laterally displaced. The distal end of the radius is wider than the mean width of the shaft. An ossified ulnar patella is present. The ulna and radius are fused distally starting at approximately the midpoint of the forearm. The metacarpal formula is IV ≥ V = III > II > I.

The hindlimb of *I*. *gunnelli* slightly differs from other Green River bats. The greater trochanter does not extend proximally as far as the femoral head, which differs from the condition seen in *I*. *index* and *O*. *finneyi*, in which the greater trochanter extends as far proximally as the level of the femoral head. The lesser trochanter is large and forms an extended flange in *I*. *gunnelli* as in *O*. *finneyi*. By contrast, the lesser trochanter is present only as a small tubercle in *I*. *index*. A third trochanter is absent in all three Green River bat taxa. The femur shaft is straight in anterior view and a femoral neck is absent. A small articulation is present between the distal ends of the tibia and fibula. The fibula is fully ossified and the proximal ends of the fibula and tibia are possibly sutured.

The first digit of the foot is shorter than the remaining digits. There is no visible contact between metatarsals of adjacent digits although intermetatarsal contact and/or facets may have been present, but are not detectable in our specimens. The shape of the proximal margin of the proximal phalanx seems to be flat in lateral view, but it is possible that this is due to taphonomic alteration. Foot digits II–V each have three phalanges.

### Phylogenetic relationships

**Fig 6:**
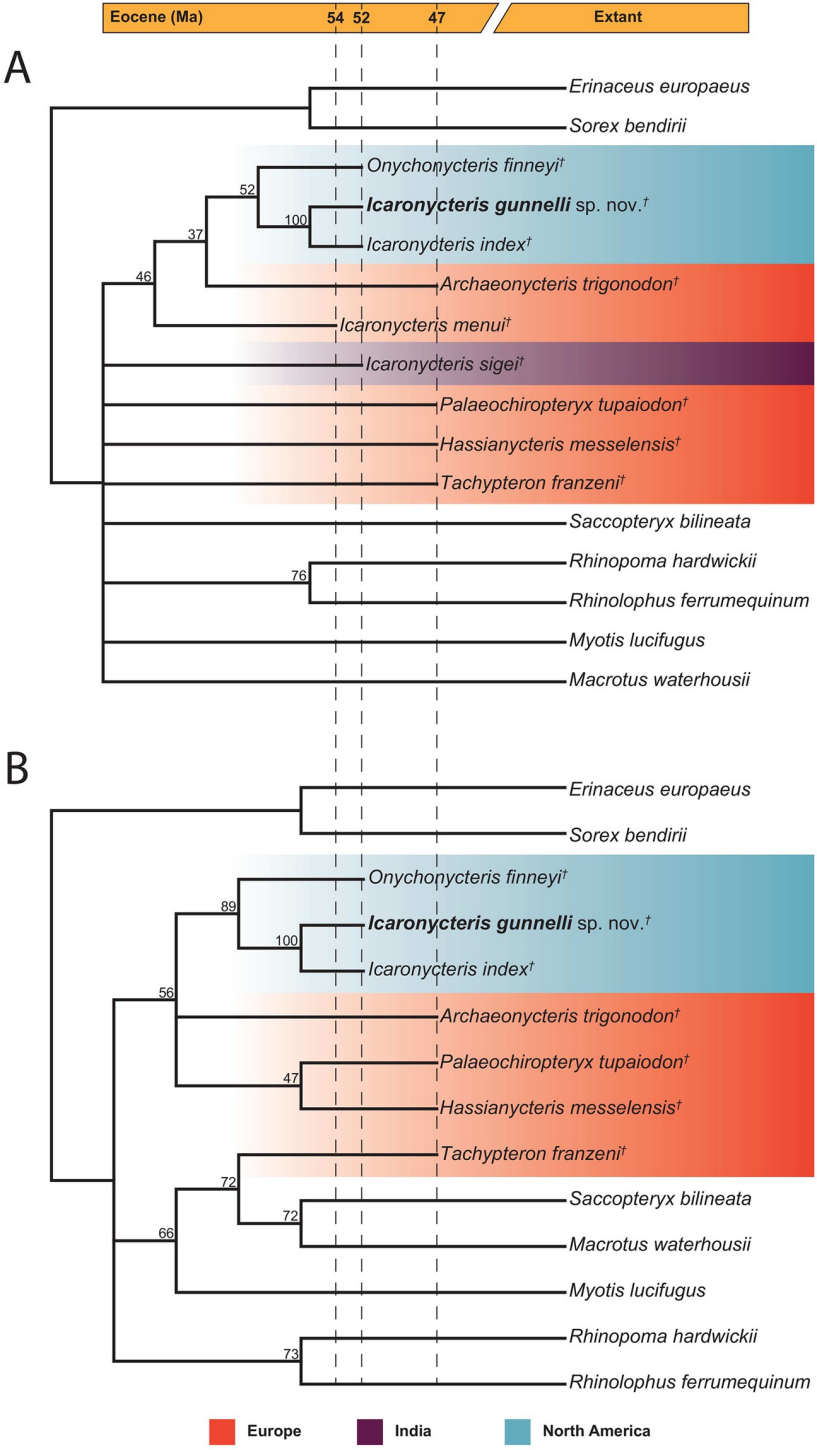
Phylogentic position of *Icaronycteris gunnelli* and other Eocene bat fossils with respect to extant bat lineages. A) Strict consensus of three most parsimonious trees of 1511 steps resulting from analyses including all taxa; B) Strict consensus of two most parsimonious trees of 1455 steps for analyses excluding *Icaronycteris*? *menui* and *Icaronycteris sigei*. Bootstrap values from 10,000 bootstrap replicates are shown above and to the left of nodes. Fossil taxa are represented by a dagger (†).

Phylogenetic analyses including all taxa resulted in three most parsimonious trees (MPTs) of 1511 steps (Fig 6A). The strict consensus tree recovered a sister relationship between *Icaronycteris gunnelli* and *Icaronycteris index* with high bootstrap support (bootstrap value 100). *Onychonycteris finneyi* fell sister to the *Icaronycteris* clade with lower support (bootstrap value 52), while *Archaeonycteris trigonodon* was recovered sister to the clade of Green River bats with low support (bootstrap value 37). *Icaronycteris menui* fell sister to the above clade in the strict consensus tree, also with low bootstap support (bootstrap value 46). The only other clade recovered in the strict consensus tree is the sister relationship between *Rhinolophus ferrumequinum* and *Rhinopoma hardwickii*, which received relatively high support (bootstrap value 76). *Tachypteron franzeni* in this analysis occurred in a polytomy with the previously mentioned clades, the remaining extant bats, *Palaeochiropteryx tupaiodon*, *Hassianycteris messelensis*, *Icaronycteris*? *menui*, and *I*. *sigei*.

Due to the fragmentary nature of both *Icaronycteris menui* and *I*. *sigei*, we conducted a second analysis omitting those taxa. This analysis resulted in two MPTs of 1420 steps each (Fig 6B). The strict consensus tree again recovers an *I*. *gunnelli* and *I*. *index* sister relationship, with *O*. *finneyi* basal to that clade. *Archaeonycteris trigonodon* falls in a polytomy to the Green River bats in this analysis, along with a clade consisting of *Palaeochiropteryx tupaiodon* and *Hassianycteris messelensis*. The *I*. *gunnelli* + *I*. *index* clade again received high bootstrap support (bootstrap value 100), while the position of *O*. *finneyi* basal to that clade also received high support (bootstrap value 89). The position of *A*. *trigonodon* basal to the Green River clade received lower support (bootstrap value 54), as did the polytomy of the Green River bats + *Archaeonycteris* plus *P*. *tupaiodon* and *H*. *messelensis* (bootstrap value 56). *Tachypteron franzeni* in this analysis fell sister to a clade consisting of the extant emballonurid *Saccopteryx bilineata* and the extant phyllostomid *Macrotus waterhousii* with moderate support (bootstrap value 72).

## Discussion

The morphology of *Icaronycteris gunnelli* unambiguously places this new taxon within *Icaronycteris* as the sister taxon of *I*. *index*. The family Icaroncyteridae as previously recognized (e.g., [27]) contained one genus and three species, with only one species (*I*. *index*) known from North America. The two other referred species are from the early Eocene of Europe and Asia: *Icaronycteris*? *menui* from France [16, 38] and *Icaronycteris sigei* from India [27]. It has always been somewhat ambiguous as to whether these latter species actually belong to the genus *Icaronycteris* in the sense of forming a clade with the type species, *I*. *index*, or simply share plesiomorphic features with that taxon. If the *Icaronycteris* (and by extension, the family Icaroncyteridae) is defined as including *I*. *sigei*, both the genus and the family can seemingly be diagnosed only by lack of apomorphies seen in other Eocene bat genera [54]. It is therefore possible that *Icaronycteris* and Icaronycteridae as thus defined might not represent a single lineage [54].

*Icaronycteris gunnelli* was placed as sister to *I*. *index* in all of our analyses with high support (Fig 6A and 6B). Both taxa are known from the same locality in the early Eocene of western North America, indicating at least a modest radiation of *Icaronycteris* in the Green River fauna. The sister group of *Icaronycteris* was found to be *Onychonycteris*, also from the Green River Formation. *Onychonycteris* was never recovered as the most basal bat, but instead consistently formed a clade with *I*. *index* and *I*. *gunnelli*. The placement of *Onychonycteris* received high support in the analysis excluding the more fragmentary taxa (bootstrap value 89; Fig 6B). This relationship—a sister-group relationship between *Icaronycteris* and *Onychonycteris*—has been recovered previously [55], but *Onychonycteris* has more frequently been recovered as the earliest diverging stem bat [12, 56, 57].

*Icaronycteris*? *menui* from the early Eocene of France was never recovered as grouping with the type species of *Icaronycteris* (*I*. *index*) but instead was placed as sister to a clade consisting of *Icaronycteris*, *Onychonycteris finneyi*, and *Archaeonycteris trigonodon* in our strict consensus tree. Relatively low bootstrap values support the above clade (bootstrap value 46; Fig 6A), with still lower support suggesting that it belongs to a clade with the North American *Icaronycteris* (bootstrap value 36). These results suggest *Icaronycteris*? *menui* likely represents a stem bat, but based on our analyses this taxon cannot be confidently assigned to the genus *Icaronycteris* or the family Icaronycteridae. We therefore state that it will be treated as Chiroptera *incertae sedis* until additional fossils or evidence emerges to allow definitive placement. Similarly, *I*. *sigei* from the early Eocene of India never grouped with other species of *Icaronycteris* in our analyses, instead occurring in a basal polytomy in our strict consensus tree (Fig 6A). Bootstrap values supporting the position of *I*. *sigei* are low, but place *I*. *sigei* either in a clade with *Saccopteryx bilineata*, *Macrotus waterhousii*, and *Tachypteron franzeni* (bootstrap value 33), sister to *M*. *waterhousii* (bootstrap value 32), or in a clade with *S*. *bilineata*, *M*. *waterhousii*, *T*. *franzeni*, and *Myotis lucifugus* (bootstrap value 31). These results suggest that *I*. *sigei* likely does not belong within *Icaronycteris* or Icaronycteridae, and may in fact be a crown bat. *Icaronycteris sigei* is currently known only from parts of lower dentition [27], and it does not seem that this material is sufficient to allow unambiguous assessment of its phylogenetic and taxonomic affinities other than to show that it does not seem to be an icaronycterid. Recovery of an upper dentition of *I*. *sigei* might help to stabilize its position within Chiroptera. Upper molars tentatively assigned to Icaronycteridae are known from the *I*. *sigei* type locality [26], but these have not been formally associated with *I*. *sigei* and thus were not included in our analysis. Pending further study, we state that *I*. *sigei* will be treated as Chiroptera *incertae sedis* until additional fossils or evidence emerges to allow definitive placement. With neither *I*.? *menui* nor *I*. *sigei* referred to *Icaronycteris*, the definitive geographic distribution of this genus (and the family, which is currently monogeneric) is thus restricted to North America.

Other relationships among stem bats recovered in our analyses differed somewhat from previously published phylogenies. *Archaeonycteris trigonodon* from Germany was recovered as sister to the *Icaronycteris* + *Onychonycteris* clade (Fig 6A) rather than sister to a clade of all bats except *Icaronycteris* and *Onychonycteris*, where it was placed in previous analyses (e.g., [12, 57]). This placement of *Archaeonycteris* received only low to moderate support (bootstrap value 37 in analyses including *I*.? *menui* and *I*. *sigei* and 54 in analyses excluding the previous taxa). Future analyses including other species of *Archaeonycteris*, *Palaeochiropteryx*, and *Hassianycteris* might better resolve relationships among these taxa.

*Palaeochiropteryx* and *Hassianycteris* are typically recovered as the closest relatives to the chiropteran crown group among stem bat lineages, with most analyses indicating that *Palaeochiropteryx* is sister to crown bats (e.g., [12, 37]). The position of both *P*. *tupaiodon* and *H*. *messelensis* in our analysis was unstable, with the pair forming part of a basal polytomy in the strict consensus including both *I*.? *menui* and *I*. *sigei* (Fig 6A). In the strict consensus tree excluding the two more fragmentary taxa, *P*. *tupaiodon* and *H*. *messelensis* were sister taxa and part of a larger clade also including *I*. *index*, *I*. *gunnelli*, *O*. *finneyi*, and *A*. *trigonodon* (Fig 6B). The most commonly recovered positions of *P*. *tupaiodon* and *H*. *messelensis* in the analysis including both fragmentary “*Icaronycteris*” taxa was a sister relationship between the two (bootstrap value 42), part of a clade including extant bats, *Tachypteron franzeni*, and *I*. *sigei* (bootstrap value 25), or part of a clade including *I*. *index*, *I*. *gunnelli*, *O*. *finneyi*, *A*. *trigonodon*, and *I*.? *menui* (bootstrap value 24). In the analysis omitting the two fragmentary taxa, the most commonly recovered relationships place *P*. *tupaiodon* and *H*. *messelensis* in a clade with *I*. *index*, *I*. *gunnelli*, *O*. *finneyi*, and *A*. *trigonodon* (bootstrap value 56), sister to each other (bootstrap value 49), in a clade with extant bats and *T*. *franzini* (bootstrap value 36), or in a clade with *I*. *index*, *I*. *gunnelli*, and *O*. *finneyi* (bootstrap value 27). There is low support for a clade consisting of the crown bats plus *H*. *messelensis* to the exclusion of *P*. *tupaiodon* (bootstrap value 19). *Tachypteron franzeni* in our analyses fell within crown Chiroptera as predicted in its description [58], but in our analysis was placed as sister to a clade consisting of *Saccopteryx bilineata* and *Macrotus waterhousii* (representing superfamilies Emballonuroidea and Noctilionoidea, respectively) rather than sister to *S*. *bilineata* as would be predicted for an emballonurid (bootstrap value 72 in analysis excluding more fragmentary taxa, 42 in analysis including all taxa). However, our placement might be the result of very limited sampling of extant taxa, so the familial relationship of *Tachypteron* with extant emballonurids as suggested by Storch et al. [58] cannot be considered refuted by our results and requires further testing with a larger taxonomic sample.

Results of our phylogenetic analyses suggest a number of potential synapomorphies in the dentition of *Icaronycteris* (= Icaronycteridae), consisting of *I*. *index* and *I*. *gunnelli*, including: (1) I1 crown undifferentiated from the root shaft, (2) upper canine lacking a lingual cingulum, (3) weak posterolateral accessory cusp on the upper canine, (4) a P4 postparacrista that does not extend to the distal margin of the tooth, (5) M1 and M2 with paraconule, (6) M1 postprotocrista becomes confluent with metacingulum distobuccally, (7) lower molars with relatively low lingual cusps, and (8) coronoid process of the dentary twice the height of the condyloid process. Character polarity in early bats is difficult to determine due to the lack of closely-related outgroups (especially outgroups with a relatively unmodified tribosphenic dentition). However, species of *Icaronycteris* have been suggested to be united solely on the basis of symplesiomorphies, clearly an undesirable situation [54]. The features listed above are not shared by most other early diverging taxa (e.g., Onychonycteridae, Archaeonycteridae) and may prove useful for identifying other members of Icaronycteridae, although we acknowledge that some of these characters may ultimately be shown to represent symplesiomorphies if more primitive bats, or close chiropteran outgroups, are discovered in the future.

### Body mass

Our reconstructions suggest that *Icaronycteris gunnelli* weighed approximately 22.5–28.9 g., similar to the body mass estimates for *Icaronycteris index* (24–27 g.; [47]). This is remarkable because *I*. *index* appears to be a slightly larger bat in terms of forearm length (43.5mm in *I*. *index* vs. 40.6mm in *I*. *gunnelli*). The overlap in our mass estimates for these taxa can perhaps be explained by the way the body mass calculations are made. Although midshaft diameter of long bones has been shown to be a better indicator of body size in extant bats than other metrics [46], it is also a parameter that is highly affected by taphonomic processes. Due to deformation or compression of the bone during fossilization, the original shape may be altered. The left and right humerus of the holotype of *I*. *gunnelli* were measured with different diameters, 1.65 and 1.81mm respectively, explaining the wide range of body size estimates for this species. The differences in measurements of the right and left humeri are doubtless a result of taphonomy since in living animals such measurements are nearly identical. It remains possible that the actual body mass of *I*. *gunnelli* did not overlap with that of *I*. *index* or that the latter species was indeed significantly larger than the former species, but we cannot rule out considerable overlap given the wide range of the body mass estimation for the former taxon.

### Stratigraphy

The published stratigraphic record of the Green River Formation regarding fossil bats is extremely limited. Often information on the exact location of where the fossil was collected—let alone details of which stratigraphic layer it came from—is lacking in species descriptions. Details of the collection locality of the holotype of *Icaronycteris index* (YPM:VPPU:018150) are not clear, with different sources reporting different information. According to archival records of the Yale Peabody Museum, the holotype of *I*. *index* was purchased from Clarence Cushman (La Barge, WY) in 1940 and was collected in 1935 from the Holland brother’s quarry, approximately 5 miles west of Kemmerer, Lincoln Co., Wyoming. However, according to Jepsen [14], the fossil was found by C. Cushman (Rangely, CO) around 1933 and presented to Princeton University in 1941, whereas Grande [41] wrote that this fossil was found at the Lewis Ranch site #2, also known as the Smith Hollow Quarry. While the exact location of the collection locality holotype of *I*. *index* thus remains confused, all three sources agree that it was discovered in the famous 18-inch layer (also referred to as “F-1” or “black-fish” layer; [41]) of the Green River Formation. While at least two other specimens of *I*. *index* have been found in this layer (UW:FV:21481a/b; FOBU:13777), other specimens come from the Sandwich Beds about one meter below the 18-inch layer.

Both known specimens of *Onychonycteris finneyi* (ROM:Palaeobiology-Vertebrate Fossils:55351A & AMNH:FM:142467) and the holotype of *Icaronycteris gunnelli* were found in the American Fossil Quarry. According to the archival records of the ROM and AMNH, the ROM specimen of *O*. *finneyi* was found 16.5 cm below the Tri-ash layers (= layers about 21 cm below the top ash of the lower sandwich beds). The AMNH specimen of *O*. *finneyi* was found below the “13-inch layers” about 91 cm above the “Red line ash” which forms the base of the sandwich beds. The holotype of *I*. *gunnelli* was discovered about 115 cm below the Tri-ash layers by Terry Rickords in 2017. The paratype of *I*. *gunnelli* was found in the bottom 60cm of the Sandwich Beds, just below the holotype of *I*. *gunnelli*. The two specimens of *I*. *gunnelli* are collected lower in the stratigraphic column than other bats mentioned above and thus represent the oldest two bat skeletons known. Interestingly, our stratigraphic analysis indicates no overlap in the temporal ranges of known Green River bat species, with the genus *Icaronycteris* including both the youngest and the oldest species (Fig 5).

Isolated teeth from Clarkforkian deposits in North America have also been referred to cf. *Icaronycteris* sp. by Gingerich [59, 60], thus potentially extending the range of *Icaronycteris* deeper in time to the late Paleocene. Other fragmentary material suggests that *Icaronycteris* may have persisted in North America until the Gardenerbuttean, an early Bridgerian interval that ended approximately 50 Mya [61]. *Icaronycteris* sp. is also mentioned from early-middle Eocene locations from Wyoming [62]. However, whether or not these specimens actually represent members of the *Icaronycteris* clade identified here (consisting of *I*. *index* and *I*. *gunnelli*) remains to be determined. In this respect, we may only be at the brink of discovering the true diversity of the early Eocene bat fauna in North America.

## Supporting information

S1 Fig CT visualizations of *Icaronycteris gunnelli* sp. nov. (holotype). A) ventral view skull; B) labial view of right dentary; C) Dorsal view skeleton; D) Occlusal view of right maxilla.
(TIF)

S1 Table Measurements of *Icaronycteris gunnelli* sp. nov. (holotype + paratype), *I*. *index* (holotype) and *Onychonycteris finneyi* (ROM + AMNH specimens) in mm. Character abbreviations: TSL—total skull length; PL—palatal length; EWCT—external width canine teeth; EWMT—external width molar teeth; PW—palatal width; POW—postorbital width; ZW—width zygomatic arch; SW—skull width; BL—body length; T—tail length; FL—femur length; FIL—fibula length; TL—tibia length; HL—humerus length; RL—radius length; Digit I—total length of digit 1; MC—metacarpal length; PP—proximal phalange length; IP—intermediate phalange length; DP—distal phalange length; LM—total length mandibula; A-m3—length lower toothrow; HCP—height coronoid process; m—lower molar; M—upper molar; p—lower premolar; P—upper premolar; c—lower canine; C—upper canine; i—lower incisor; I—upper incisor; Fdigit—total length of foot digit; FMT—metatarsal length; FPP—foot proximal phalange length; FIP—foot intermediate phalange length; FDP—foot distal phalange length; CCL—calcar length.
(DOCX)

S1 File Calculations of body mass. (DOCX)

S1 Appendix Bat specimens and casts examined as part of this study. Taxa not listed were coded from the literature. Fossil taxa are represented by a dagger (†).
(XLSX)
