# Supplementary figures and images for: The oldest known bat skeletons and their implications for Eocene chiropteran diversification
Source: PLoS One. 2023 Apr 12;18(4):e0283505. doi: 10.1371/journal.pone.0283505 (PMC10096270; doi:10.1371/journal.pone.0283505)

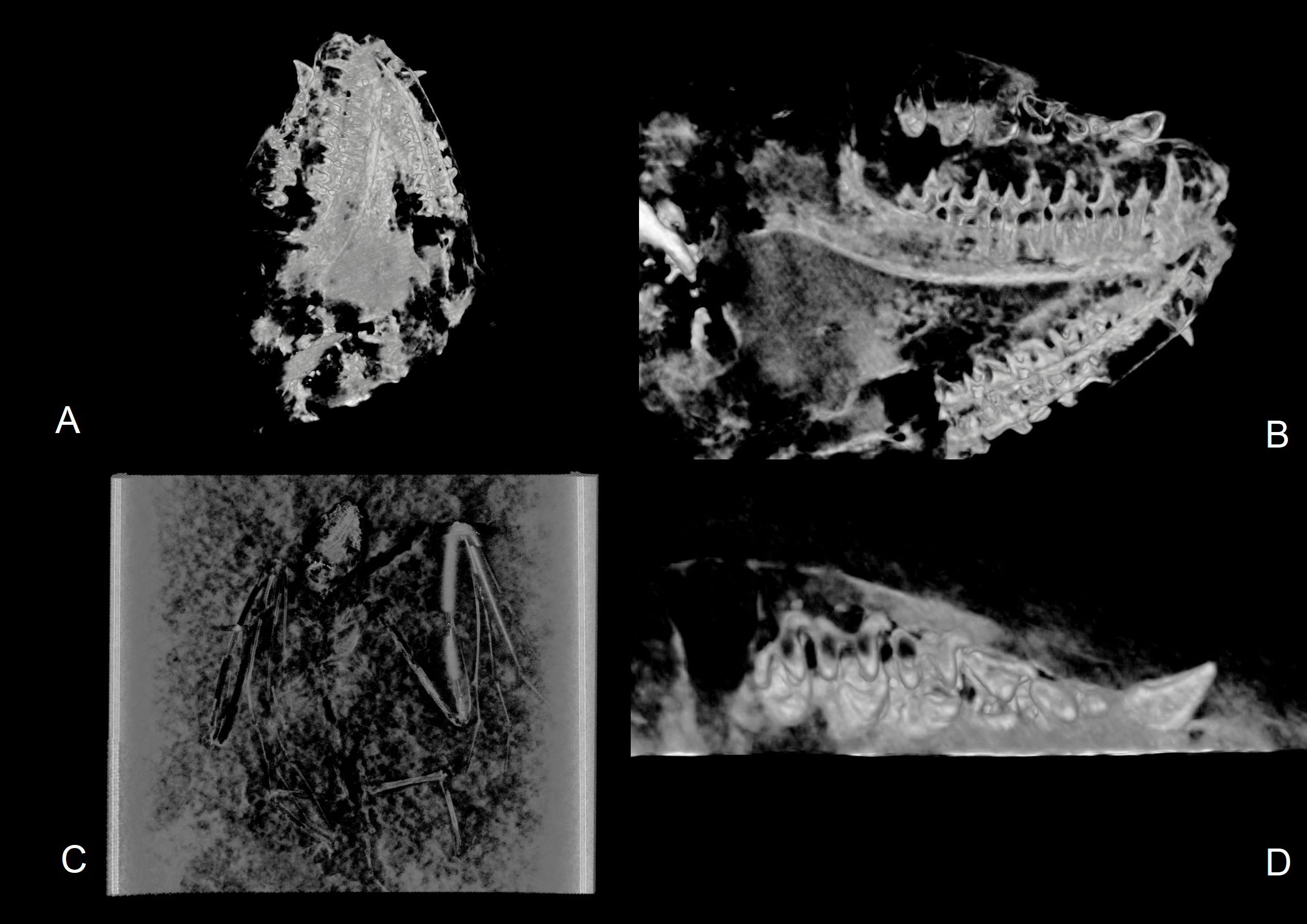

Supplement: S1 Fig — A) ventral view skull; B) labial view of right dentary; C) Dorsal view skeleton; D) Occlusal view of right maxilla. (TIF) [file pone.0283505.s001.tif]
