# Supplementary material for: The oldest known bat skeletons and their implications for Eocene chiropteran diversification
Source: PLoS One. 2023 Apr 12;18(4):e0283505. doi: 10.1371/journal.pone.0283505 (PMC10096270; doi:10.1371/journal.pone.0283505)
Supplement: S1 Table — Character abbreviations: TSL—total skull length; PL—palatal length; EWCT—external width canine teeth; EWMT—external width molar teeth; PW—palatal width; POW—postorbital width; ZW—width zygomatic arch; SW—skull width; BL—body length; T—tail length; FL—femur length; FIL—fibula length; TL—tibia length; HL—humerus length; RL—radius length; Digit I—total length of digit 1; MC—metacarpal length; PP—proximal phalange length; IP—intermediate phalange length; DP—distal phalange length; LM—total length mandibula; A-m3—length lower toothrow; HCP—height coronoid process; m—lower molar; M—upper molar; p—lower premolar; P—upper premolar; c—lower canine; C—upper canine; i—lower incisor; I—upper incisor; Fdigit—total length of foot digit; FMT—metatarsal length; FPP—foot proximal phalange length; FIP—foot intermediate phalange length; FDP—foot distal phalange length; CCL—calcar length. (DOCX) [file pone.0283505.s002.docx]

|  | *Icaronycteris gunnelli* (Holotype) | *Icaronycteris gunnelli* (Paratype) | *Onychonycteris finneyi* | *Onychonycteris finneyi* | *Icaronycteris index* (Holotype) |
| --- | --- | --- | --- | --- | --- |
|  | **AMNH.FM.145747** | **ROM 52666** | **ROM 55351A** | **AMNH.FM.142467** | **YPM-PU 18150** |
| **TSL** | 17.53 | 19.7 | 22.6 | 23.7 | 20.1 |
| **PL** | (---) | (---) | (---) | (---) | (---) |
| **EWCT** | 4.67 | (---) | 5.28 | (---) | ~4.0 |
| **EWMT** | (---) | (---) | 7.61 | (---) | ~7.8 |
| **PW** | (---) | (---) | (---) | (---) | (---) |
| **POW** | (---) | (---) | (---) | (---) | 6.84 |
| **ZW** | 13.04 | (---) | 14.96 | (---) | 12.55 |
| **SW** | 9.94 | (---) | 10.18 | 10.22 | 7.22 |
| **BL** | 43.2 | 48 | 55.5 | 62.6 | 50.5 |
| **T** | 41 | 47.3 | 56.9 | 63.8 | 52.4 |
| **FL** | 16.9 | 17.3 | 26.6 | 26.3 | 19.8 |
| **FIL** | 15.83 | 15.29 | 24.84 | 24.45 | 18.4 |
| **TL** | 15.73 | 17.1 | 24.6 | 25.5 | 18.3 |
| **HL** | 30.94 | 30.2 | 35.2 | 36.4 | 34.3 |
| **RL** | 40.56 | 41.6 | 45.8 | 45.4 | 48 |
| **Digit I** | 8.34 | (---) | (---) | 11.3 | 11.9 |
| **MC I** | 2.72 | (---) | (---) | 4.9 | 3.5 |
| **PP I** | 3.37 | (---) | (---) | 4 | 5.7 |
| **DP I** | 2.25 | 1.9 | 2.6 | 2.4 | 2.7 |
| **Digit II** | 31.27 | 33 | 35.1 | 37.1 | 39.8 |
| **MC II** | 22.31 | 23.2 | 21.2 | 21.5 | 28.5 |
| **PP II** | 3.38 | 4.1 | 5.2 | 6 | 4.9 |
| **IP II** | 3.99 | 4.5 | 6.4 | 6.7 | 4.9 |
| **DP II** | 1.59 | 1.2 | 2.3 | 2.9 | 1.5 |
| **Digit III** | 58.71 | 65.9 | 64.2 | 66.9 | 70.2 |
| **MC III** | 32.38 | 36 | 30.2 | 30.2 | 40.1 |
| **PP III** | 10 | 10.4 | 13.8 | 15.3 | 10.9 |
| **IP III** | 16.03 | 19.2 | 18.3 | 20.1 | 18.8 |
| **DP III** | 0.3 | 0.3 | 1.9 | 1.3 | 0.4 |
| **Digit IV** | 56.95 | 62 | 61.8 | 64.3 | 66.7 |
| **MC IV** | 33.29 | 35.6 | 30.7 | 31.9 | 39 |
| **PP IV** | 9.55 | 10.6 | 13.6 | 14.5 | 11.5 |
| **IP IV** | 13.81 | 15.8 | 16.2 | 17 | 16.1 |
| **DP IV** | 0.3 | 0.2 | 1.3 | 0.9 | *0.3? |
| **Digit V** | 52.57 | 57.7 | 63.1 | 64.5 | 60.6 |
| **MC V** | 32.89 | 36.6 | 34.7 | 34.9 | 38 |
| **PP V** | 8.89 | 8.9 | 14 | 14.7 | 10.1 |
| **IP V** | 10.39 | 12 | 13.5 | 14.2 | 12.2 |
| **DP V** | 0.4 | 0.2 | 0.9 | 0.7 | 0.3 |
| **LM** | 11.83 | 13.18 | 16.39 | 16.17 | 15.3 |
| **A-m3** | 4.16 | 4.63 | 6.37 | 6.38 | 6.2 |
| **HCP** | 6.19 | 5.3 | (---) | 5.74 | (---) |
| **m1 - m3** | 3.92 | 3.76 | 4.74 | 4.45 | 4.5 |
| **p4 - m3** | 4.84 | 4.87 | (---) | 5.8 | *5.9 |
| **p3 - m3** | 5.71 | 5.71 | (---) | 6.39 | *6.8 |
| **p2 - m3** | 6.44 | 6.48 | (---) | 7.35 | 7.7 |
| **c1 - m3** | 7.41 | 7.55 | 8.08 | 8.54 | 8.3 |
| **i1 - m3** | 8.18 | 8.32 | 9.89 | 9.39 | 9.3 |
| **m3** | 1 | 1.13 | 1.43 | 1.4 | 1.5 |
| **m2** | 1.34 | 1.24 | 1.45 | 1.48 | 1.5 |
| **m1** | 1.37 | 1.5 | 1.45 | 1.48 | 1.5 |
| **M1 - M3** | 3.31 | 3.36 | 4.76 | 4.39 | 3.7 |
| **P4 - M3** | 4.18 | 4.55 | 5.76 | 5.42 | (---) |
| **P3 - M3** | 4.95 | 5.54 | 6.41 | 6.54 | (---) |
| **P2 - M3** | 5.64 | 6.52 | 7.23 | 7.32 | (---) |
| **C1 - M3** | 6.59 | 7.45 | 8.92 | 9.07 | (---) |
| **I1 - M3** | 8.03 | 8.79 | 9.85 | 9.94 | (---) |
| **M3** | 0.92 | 1 | 1.43 | 1.5 | 1.3 |
| **M2** | 1.18 | 1.16 | 1.82 | 1.77 | 1.4 |
| **M1** | 1.2 | 1.2 | 1.94 | 1.95 | 1.3 |
| **Fdigit I** | 6.28 | 7.23 | 7.07 | 7.4 | 8.7 |
| **FMT I** | 2.18 | 2.07 | 2.92 | 2.7 | 2.8 |
| **FPP I** | 2.44 | 3.48 | 2.64 | 3.2 | 3.4 |
| **FDP I** | 1.66 | 1.68 | 1.51 | 1.5 | 2.5 |
| **Fdigit 2** | 7.16 | 8.32 | 9.04 | 9.8 | 10.4 |
| **FMT II** | 2.19 | 2.4 | 2.84 | 2.9 | 3.2 |
| **FPP II** | 1.66 | 2.28 | 2.42 | 2.9 | 2.4 |
| **FIP II** | 1.62 | 2.01 | 2.06 | 2.1 | 2.5 |
| **FDP II** | 1.69 | 1.63 | 1.72 | 1.9 | 2.3 |
| **Fdigit 3** | 7.35 | 8.42 | 9.62 | 10.9 | 10.6 |
| **FMT III** | 2.22 | 2.5 | 3 | 2.9 | 3.3 |
| **FPP III** | 1.63 | 2.28 | 2.44 | 3.3 | 2.5 |
| **FIP III** | 1.75 | 2.01 | 2.29 | 2.8 | 2.5 |
| **FDP III** | 1.75 | 1.63 | 1.89 | 1.9 | 2.3 |
| **Fdigit IV** | 7.41 | 8.46 | 9.9 | 9.7 | 10.7 |
| **FMT IV** | 2.25 | 2.5 | 3.02 | 2.9 | 3.4 |
| **FPP IV** | 1.69 | 2.28 | 2.54 | 2.6 | 2.5 |
| **FIP IV** | 1.75 | 1.85 | 2.4 | 2.3 | 2.7 |
| **FDP IV** | 1.72 | 1.83 | 1.94 | 1.9 | 2.1 |
| **Fdigit V** | 7.57 | 5.96 | 9.29 | 9.2 | 10.5 |
| **FMT V** | 2.19 | 2.68 | 3.06 | 2.7 | 3.1 |
| **FPP V** | 1.81 | 2.28 | 2.3 | 2.3 | 2.6 |
| **FIP V** | 1.91 | 2.07 | 2.3 | 2.3 | 2.7 |
| **FDP V** | 1.66 | 1.61 | 1.63 | 1.9 | 2.1 |
| **CCL** | (---) | (---) | 10.94 | (---) | (---) |
