# Supplementary material for: The oldest known bat skeletons and their implications for Eocene chiropteran diversification
Source: PLoS One. 2023 Apr 12;18(4):e0283505. doi: 10.1371/journal.pone.0283505 (PMC10096270; doi:10.1371/journal.pone.0283505)
Supplement: S1 File — (DOCX) [file pone.0283505.s003.docx]

*General Model: log_10_ D = log_10_ b0 + b1 * log_10_ M + error*

*Input data: D = 1.652 mm hence log_10_ D = 0.21801*

*Model parameters for humerus (from Table 16.1 of book chapter 16, Giannini et al. 2012)*

*log_10_ b0 = -0.273*

*b1 = 0.363*

*Solving Model to calculate point estimate M (without error term):*

*0.21801 = -0.273 + 0.363 * log_10_ M
(0.21801 + 0.273) / 0.363 = log_10_ M (this as in eq. 16.1 of book chapter 16, Giannini et al. 2012)*

*log_10_ M = 1.35264*

*M = 22.524 (g)*
